# Brown rats (*Rattus norvegicus*) as potential reservoirs of *Enterocytozoon bieneusi* in Heilongjiang Province, China: high prevalence, genetic heterogeneity, and potential risk for zoonotic transmission

**DOI:** 10.3389/fvets.2024.1426384

**Published:** 2024-07-25

**Authors:** Yanyan Jiang, Shanshan Zhou, Zhongying Yuan, Xinyu Hu, Zhen Li, Yaxue Wang, Yujuan Shen, Jianping Cao

**Affiliations:** ^1^National Institute of Parasitic Diseases, Chinese Center for Disease Control and Prevention (Chinese Center for Tropical Diseases Research), National Key Laboratory of Intelligent Tracking and Forecasting for Infectious Diseases, NHC Key Laboratory of Parasite and Vector Biology, National Center for International Research on Tropical Diseases, WHO Collaborating Centre for Tropical Diseases, Shanghai, China; ^2^School of Global Health, Chinese Center for Tropical Diseases Research, Shanghai Jiao Tong University School of Medicine, Shanghai, China

**Keywords:** *Enterocytozoon bieneusi*, *Rattus norvegicus*, zoonotic, genotype, China

## Abstract

**Introduction:**

*Enterocytozoon bieneusi*, an obligatory intracellular fungus, is prevalent among animals and humans. Due to their close interaction with humans and their extensive regional distribution, brown rats (*Rattus norvegicus*) are important pathogen reservoirs. To assess the zoonotic transmission potential of *E. bieneusi*, a molecular investigation was conducted on 817 *R. norvegicus* from four cities in Heilongjiang Province, China.

**Methods:**

A total of 817 R. norvegicus were collected from four cities in Heilongjiang Province, China. The genotyping of E. bieneusi was conducted through PCR amplification of the small subunit ribosomal RNA (SSU rRNA)’s internal transcribed spacer (ITS) segments. Phylogenetic and similarity analyses were used to examine zoonotic potential and genetic characteristics of the E. bieneusi-positive specimens.

**Results:**

Among the 817 *R. norvegicus,* the total infection rate was 33.3% (272/817). Seventy-five genotypes were identified, including 14 known genotypes D (*n* = 167), A (*n* = 15), HLJ-CP1 (*n* = 12), WR8 (*n* = 6), EbpC (*n* = 2), BEB6 (*n* = 1), CS-4 (*n* = 1), CHPM1 (*n* = 1), Henan-II (*n* = 1), HNH-22 (*n* = 1), HNH-25 (*n* = 1), I (*n* = 1), JLD-XI (*n* = 1), SDD5 (*n* = 1), and 61 novel genotypes designated as SHWR1 (*n* = 10), SYSWR1 (*n* = 2), and SHWR2 to SHWR17, SYSWR2 to SYSWR36 and QTHWR1 to QTHWR8 (*n* = 1, each). Moreover, 10 samples exhibited mixed genotype infections, including D + A (*n* = 3), D + EbpC (*n* = 1), D + HLJ-CP1 (*n* = 1), D + SHWR1 (*n* = 1), D + SHWR16 (*n* = 1), D + SHWR17 (*n* = 1), SDD5 + WR8 (*n* = 1), and CS-4 + SYSWR36 (*n* = 1). Phylogenetic analysis grouped the genotypes into three main groups: group 1 (*n* = 67), group 2 (*n* = 5), and group 9 (*n* = 3).

**Discussion:**

The high prevalence and genetic diversity of *E. bieneusi* in Heilongjiang Province’s *R. norvegicus* imply that these animals spread the pathogen. The *R. norvegicus* that *E. bieneusi* carries can spread zoonotic disease, making it a serious hazard to the local human population. Therefore, it is imperative to raise awareness about the dangers posed by *R. norvegicus* and implement measures to reduce their population to prevent environmental contamination.

## Introduction

Microsporidia, a vast and diverse group of intracellular fungi, encompass more than 1,500 species. While most of these species are innocuous to human beings, 17 species have been identified as human pathogens (1). *Enterocytozoon bieneusi* is the most common cause of human microsporidial infections, making up more than 90% of cases (2). The spore, which is the infectious form of *E. bieneusi*, is expelled from the feces of the host and dispersed into the environment, thereby ensuring its widespread presence (3). Transmission of spores to humans can occur via direct contact, including person-to-person or animal-to-person transmission, as well as indirectly through contaminated food or water sources (4). Once contaminated, pathogenic spores may spread easily among individuals and other animals, exacerbating environmental contamination. There is an example of a food-borne outbreak linked to *E. bieneusi* infection, as well as a reported large outbreak of waterborne diseases caused by lake contamination (5, 6). Regular surveillance of *E. bieneusi* infections in both human and animal populations is crucial for implementing preventive interventions to reduce the spread of these diseases and minimize the probability of outbreaks. Accurate identification of the causal agent is essential for implementing effective monitoring and control strategies.

The advent of molecular typing tools provides the possibility for accurate identification of *E. bieneusi*. Currently, the main technique used to identify *E. bieneusi* is by sequencing analysis of the internal transcriptional spacer (ITS) within the small subunit ribosomal RNA (*SSU rRNA*) gene of this pathogen (7). The combination of homology and phylogenetic analysis provides an important basis for assessing genotype transmission across species and host adaptability characteristics among different hosts (8). Recently, researchers have identified around 900 different genotypes of *E. bieneusi*. Out of them, more than 150 genotypes are found only in humans, around 700 genotypes are found only in animals, and 67 genotypes are shared by both species (9–11). Notably, the total number of genotypes continues to grow as more genotypes are discovered, along with a concurrent increase in the number of zoonotic genotypes. For instance, in 2011, there were 93 genotypes: 34 exclusive to humans, and 11 shared between humans and various animals (12). The overall number of genotypes had expanded nine times by 2021, while the number of genotypes associated with zoonotic diseases had increased six times (9). If this pattern continues, thousands of novel genotypes might be documented in the next decade. Evolutionary analysis has also been considered as another effective means of assessing the possibility of zoonotic transmission. Currently, the genotypes that have been found are categorized into 15 distinct classifications (8, 13). The majority of these genotypes, which infect humans or animals, belong to the first and second groups. This indicates their significance in the formation of possible clusters of zoonotic diseases. The remaining 13 clusters are mainly present in specific hosts and wastewater (13).

In humans, genotypes D, EbpC, Type IV, and EbpA are the most common, and all have a broad host range (3, 9). Thus, there is great interest in determining the sources of infection for these zoonotic genotypes. Investigation suggests that these genotypes are not only widespread in humans and animals but also frequently identified in aqueous environments (3). This implies that it is necessary to develop comprehensive health strategies to tackle the high occurrence of *E. bieneusi*. Given that they are freely roaming and extensively dispersed, wild rodents have become a crucial connection between humans, animals, and the environment. *E. bieneusi* has been documented in at least 44 rodent species across 25 studies which conducted in China, the Czech Republic, Iran, Japan, Peru, Poland, Slovakia, and the United States (11, 14, 15). At present, there are 117 genotypes of *E. bieneusi* known to exist in rodents, including at least 18 zoonotic genotypes. The most common genotype is D, Type IV, which is similar to the prevalence in humans (9, 11, 14, 15). This overlap underscores the crucial role rodents play in transmitting *E. bieneusi* to humans, emphasizing the need to incorporate rodent control into the eradication efforts against this pathogen.

China has become an influential player in molecular epidemiology data regarding *E. bieneusi* in wild rodents. The research carried out in at least 13 provinces, has yielded vital information on ways in which *E. bieneusi* is transmitted and the sources of its infection (Table 1) (11, 13–20, 22–24). However, significant data gaps persist in certain regions of China, particularly in regions such as Heilongjiang, where the presence of *E. bieneusi* in humans and other animals has been widely reported (20, 25–28). It would be advantageous to monitor rodents in these locations to get insight into the origins and means of infection transmission among humans and other animals. Therefore, the objective of this study was to ascertain the infection rates and genotype composition of *E. bieneusi* carried by wild rodents in Heilongjiang Province, China, ultimately evaluating the zoonotic transmission risk through homology and phylogenetic analysis.

**Table 1:** Occurrence and distribution of *Enterocytozoon bieneusi* genotypes in wild rodents in China by Region.

Region	Positive no./Examined no. (%)	Genotypes (*n*)	Reference
Chongqing	39/111 (35.1)	D (14), Type IV (K) (8), PigEBITS7 (22), Peru8 (2), CQR-1 (10), CQR-2 (15), CQR-3 (1), GDR-1 (2), GDR-2 (1), GDR-3 (1)	(16)
Guangdong	37/117 (31.6)		
Gansu	50/498 (10.0)	ZY37 (27), YAK1 (17), SN45 (1), XH47 (1), ZY83 (1), HN39 (1), HN96 (1), YAK1 (1)	(17)
Guangxi	5/74 (6.8)	GXM1 (5)	(18)
Hainan	69/369 (18.7)	D (24), PigEbITS7 (19), HNR-VII (15), Type IV (4), HNR-III (2), Peru 8 (1), EbpA (1), ESH-02 (1), HNR-I (1), HNR-II (1)	(19)
Heilongjiang	34/373 (9.1)	EbpB (2), SCC-1 (1), SCC-2 (3), NESQ1 (2), HLJ-CP1 (1), EbpB (1), D (17), Peru6 (2), EbpC (1), HLJC1 (2), HLJC2 (1)	(13, 18, 20)
Henan	8/199 (4.0)	CHG14 (CD6) (3), BEB6 (2), D (2), CHG2 (1)	(21)
Jiangxi	7/35 (20.0)	CHN4 (7)	(22)
Qinghai	4/98 (4.1)	Zokor genotype 1 to Zokor genotype 4 (one each)	(23)
Shandong	4/227 (1.8)	NCF2 (1), SDR1(1), D (2)	(18)
Shanxi	20/53 (37.7)	EbpA (3), EbpC (7), D (9), XJP-II (1)	(18)
Xinjiang	76/334 (22.8)	XJHT3 (48), BEB6 (14), XJHT2 (7), XJHT4 (4), CHG3 (1), YAK1 (1), GX2 (1)	(24)
Zhejiang	70/489 (24.4)	HNR-IV (19), EbpC (12), D (6), EbpA (3), WZR-VIII (5), SHW7 (2), ZJR7 (2), Henan-III (1), HNHZ-II (1), K (1), ZJR1 (1), ZJR2 (1), ZJR3 (1), ZJR4 (1), ZJR5 (1), ZJR6 (1), HNP-II (1), WZR-IX to WZR-XII (one each), WZR-I to VII (one each)	(11, 18)

## Materials and methods

### Ethics statement

All rodents included in the current study underwent capture and humane euthanasia procedures that strictly adhered to the Chinese Laboratory Animal Administration Act (2017) and the National Institutes of Health’s (NIH, 2020) guidelines for euthanasia of rodents using carbon dioxide (CO_2_). No rodent species that were endangered or protected were found in the regions that were researched. The objectives and procedures of the present study were reviewed and approved by the National Institute of Parasitic Diseases, Chinese Center for Disease Control and Prevention, China (Reference no. IPD-2021-21).

### Sampling sites and sample collection

**Figure 1:**
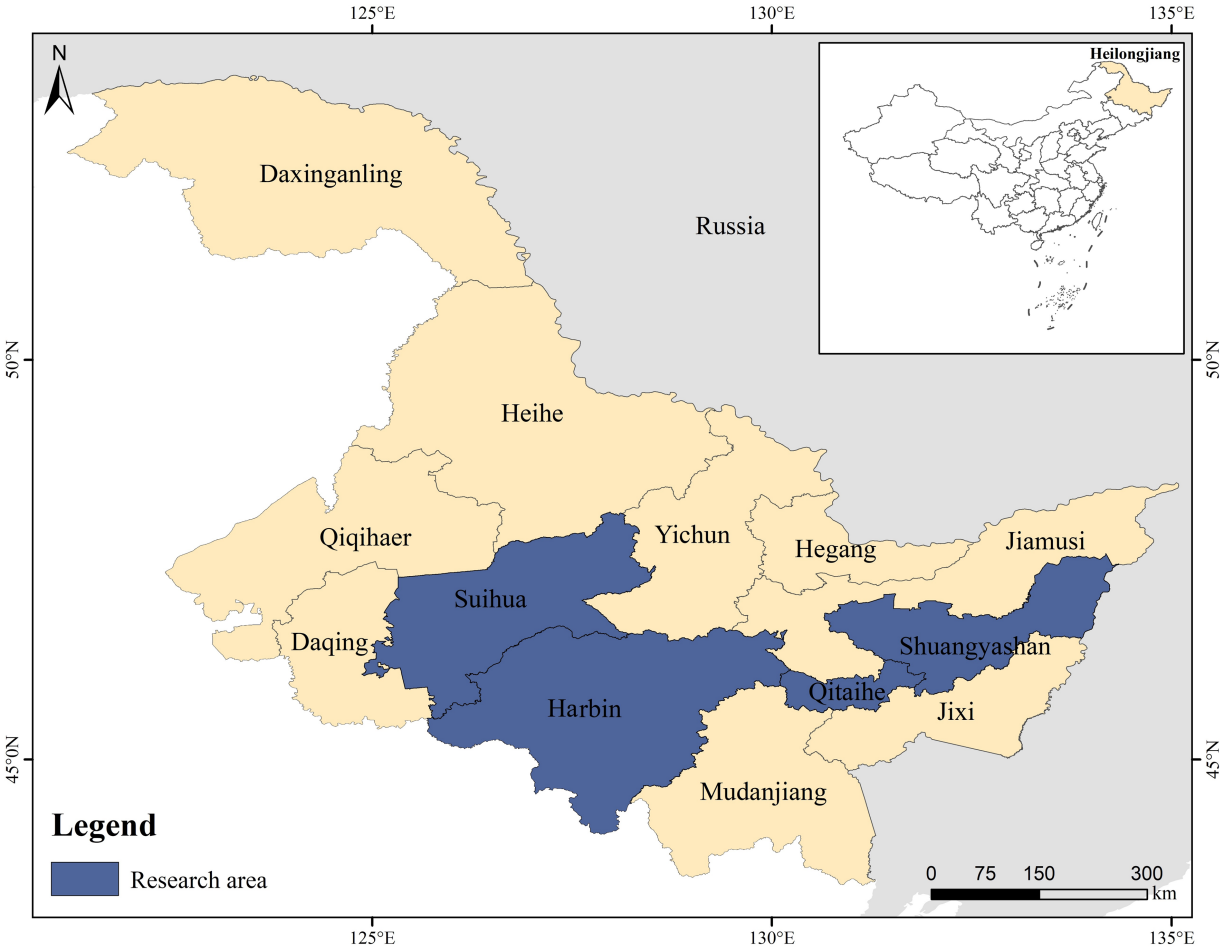
A map that illustrates the locations where *Rattus norvegicus* was sampled in Heilongjiang Province, China.

Between November 2022 and December 2023, a total of 2,000 traps were set up across residential communities in rural areas of Suihua, Qitaihe, Shuangyashan, and Harbin in Heilongjiang Province of China (Figure 1). Each trap was baited with sunflower seeds and peanut/sesame butter to attract the rodents. Throughout the study, a total of 910 rodents were captured, of which 817 were brown rats (*Rattus norvegicus*). This prompted us to focus solely on this species for the subsequent investigations. The captured brown rats were euthanized by CO_2_ inhalation. After euthanasia, the animals were transferred to the laboratory while being kept at low temperatures. Fecal samples were carefully taken from the intestinal and rectal areas of each rodent in the biosafety cabinets, following Level 2 containment protocols. Subsequently, these samples were stored in frozen conditions at a temperature of −80°C to maintain their integrity for subsequent analysis.

### Extraction of genome DNA

For DNA extraction, each fecal sample was washed with distilled water using centrifugation at room temperature for 10 min at a speed of 1,500×*g*. Genomic DNA was extracted from approximately 200 mg of each processed fecal specimen using the QIAamp DNA Stool Mini Kit (QIAgen, Hilden, Germany) according to the manufacturer’s instructions. DNA was eluted in 200 μl of ATE buffer and was stored at −80°C until required for polymerase chain reaction (PCR)-based analyses.

### PCR amplification

The *R. norvegicus* was identified at the species level by amplification of a 762 bp region of the partial mitochondrial cytochrome b (*Cytb*) gene from fecal DNA using PCR (29). Moreover, each of the DNA specimens was analyzed for *E. bieneusi* by PCR amplification of the partial *SSU rRNA* gene (fragment: approximately 390 bp; containing 243 bp of the ITS region), with the primers and the cycle parameters being described previously by Buckholt et al. (30). The PCR reactions were performed using 2 × TransTaq®-T PCR SuperMix (+dye) from TransGen Biotech Co., Beijing, China. A negative control, devoid of any DNA, was included in all PCR experiments. Each DNA sample underwent two replicated PCR reactions to identify and genotype *E. bieneusi*. The final PCR results were subjected to electrophoresis using a 1.5% agarose gel stained with GelStrain from TransGen Biotech Co., Beijing, China. The gel was then examined, photographed, and recorded using a Gel Doc ™ XR + Imaging System from Bio-Rad, USA.

### DNA sequence analysis

All the final PCR products of the expected size were sequenced with their respective secondary PCR primers in Shanghai Saiheng Biotechnology Company Limited (Shanghai, China) by an ABI 3730XL Genetic Analyzer (Applied Biosystems, Foster City, CA). To verify sequence accuracy, bidirectional sequencing was employed. The Chromas Pro 1.5 software (Technelysium, Pty, Ltd.) was used to edit the raw sequences used in this study. The sequences were then aligned with each other and reference sequences were downloaded from the GenBank database using the Basic Local Alignment Search Tool (BLASTl; http://blast.ncbi.nlm.nih.gov/Blast.cgi) and MEGA7^1^ to ascertain the genotypes of *E. bieneusi*. The two PCR products of the expected size were both sequenced from the same sample to make sure accuracy. When the sequences obtained were different, these samples were designed as mixed infection by re-sequencing to confirm the accuracy of the sequences. If the sequences obtained were identical to those published in GenBank, they were considered known genotypes and given the first published name. If not, they were deemed as novel genotypes. To ensure accuracy, two distinct PCR amplicons from the identical DNA preparation were sequenced in the event that the acquired nucleotide sequences deviated from the published ones, including any variations, insertions, or deletions in the nucleotide sequence. Following the established nomenclature scheme, the 243 base pairs of the ITS gene section of the rRNA gene were used to identify each genotype of *E. bieneusi* (7).

### Phylogenetic analysis

Using the program MEGA 7,^2^ a neighbor-joining (NJ) tree of the ITS region of all the nucleotide sequences was created to identify the phylogenetic groups and evaluate the genetic relationship and zoonotic potential of novel genotypes of *E. bieneusi* obtained in this study to the known ones. Evolutionary distances among the nucleotide sequences of the ITS region of the *SSU rRNA* gene were calculated using the Kimura-2-parameter algorithm. The reliability of branches in trees was assessed by the bootstrap analysis with 1,000 replicates.

### Statistical analyses

Statistical analyses were performed using the Statistical Package for the Social Sciences (SPSS) version 22.0, a software developed by SPSS Inc. in Chicago, Illinois, USA. The chi-square test was utilized to assess the prevalence of *E. bieneusi* in *R. norvegicus* across various regional groups, and statistical significance was determined when the *p*-values were below 0.05.

### Nucleotide sequence accession numbers

The nucleotide sequences obtained in this study have been deposited in the GenBank database and can be accessed using accession numbers ranging from PP719220 to PP719286.

## Results

### The occurrence rate of *E. bieneusi* in brown rats

During this study, 817 *R. norvegicus* were screened based on morphological characteristics. Following that, a sequential examination of the partial *Cytb* gene was carried out to determine the molecular identity of all the animals. Of these, 385 were sampled from Suihua, 210 from Qitaihe, 200 from Shuangyashan, and 22 from Harbin (Table 2). Moreover, analysis of the ITS region of the *SSU rRNA* gene revealed the presence of *E. bieneusi* in 33.3% of the brown rats (272/817) sampled across multiple regions. Specifically, the infection rates were 27.5% (106/385) in Suihua, 29.5% (62/210) in Qitaihe, 45.5% (91/200) in Shuangyashan, and 59.1% (13/22) in Harbin (Table 2). These results imply that the infection rates of brown rats from various geographic locations vary significantly (*χ*^2^ = 27.1, df = 3, *p* < 0.001).

**Table 2:** Occurrence rate of *E. bieneusi* in wild rodents in Heilongjiang Province.

Sampling site^*^	Positive no. /Examined no. (%)	Genotype (*n*)
Suihua	106/385 (27.5)	D (69), HLJ-CP1 (7), WR8 (5), A (4), D + A (1), D + HLJ-CP1 (1), D + SHWR1 (1), D + SHWR16 (1), D + SHWR17 (1), SDD5 + WR8 (1), SHWR1 to SHWR15 (one each)
Qitaihe	62/210 (29.5)	D (38), A (8), HLJ-CP1 (3), D + A (2), BEB6 (1), HNH-25 (1), SHWR1 (1), QTHWR1 to QTHWR8 (one each)
Shuangyashan	91/200 (45.5)	D (40), SHWR1 (7), SYSWR1 (2), CHPM1 (1), EbpC (1), Henan-II (1), HLJ-CP1 (1), HNH-22 (1), I (1), JLD-XI (1), CS-4 + SYSWR36 (1), SYSWR2 to SYSWR35 (one each)
Harbin	13/22 (59.1)	D (12), D + EbpC (1)
Total	272/817 (33.3)	D (159), A (12), HLJ-CP1 (11), SHWR1 (9), WR8 (5), D + A (3), SYSWR1 (2), BEB6 (1), HNH-25 (1), Henan-II (1), HNH-22 (1), I (1), JLD-XI (1), CHPM1 (1), EbpC (1), D + EbpC (1), D + HLJ-CP1 (1), D + SHWR1 (1), D + SHWR16 (1), D + SHWR17 (1), SDD5 + WR8 (1), CS-4 + SYSWR36 (1), SHWR2 to SHWR15 (one each), SYSWR2 to SYSWR35 (one each), QTHWR1 to QTHWR8 (one each)

* There was a significant difference in the infection rates of brown rats from various geographic locations.

### Genetic characterizations and genotype distribution of *E. bieneusi*

**Figure 2:**
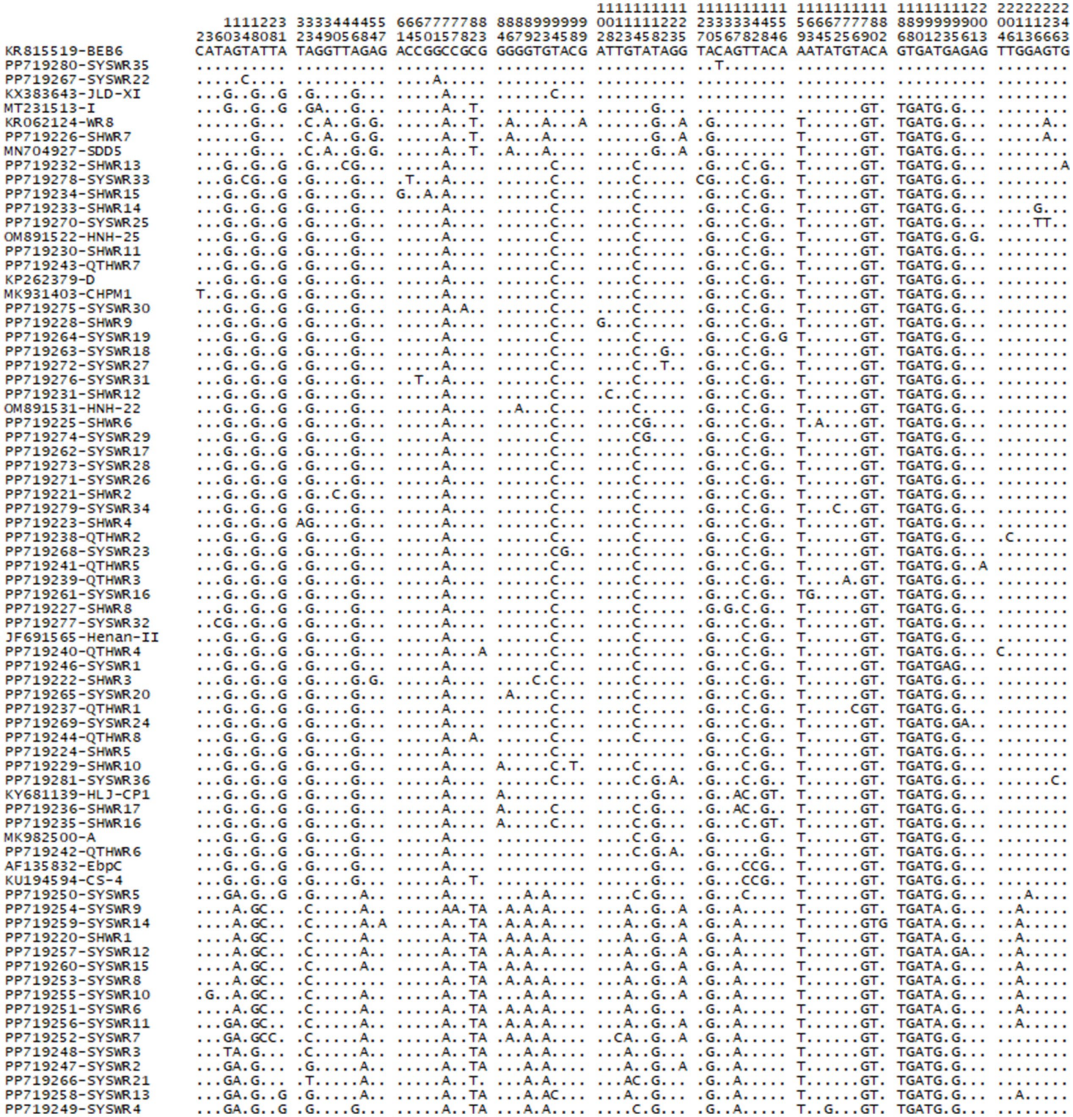
Variation among the ITS sequences of genotypes identified in the present.

From a total of 272 *E. bieneusi*-positive specimens, 75 representative sequences were obtained, encompassing 75 genotypes. Among these genotypes, 14 were known (A, BEB6, CHPM1, CS-4, D, EbpC, Henan-II, HJL-CP1, HNH-22, HNH-25, I, JLD-X1, SDD5, and WR8), while 61 were novel, designated as SHWR1 to SHWR17, QTHWR1 to QTHWR8, and SYSWR1 to SYSWR36. These genotypes showed significant genetic diversity, with 88 polymorphic sites identified (Figure 2). Of the genotypes identified, genotype D was the most prevalent, encompassing 61.4% (167/272) of the total specimens. Genotype A contributed 5.5% (15/272), genotype HLJ-CP1 accounted for 4.4% (12/272), genotype SHWR1 represented 3.7% (10/272), genotype WR8 contributed 2.2% (6/272), genotypes SYSWR1 and EbpC each contributed 0.7% (2/272), while the remaining genotypes were each represented by a single specimen. Notably, genotype D exhibited the widest geographical distribution, being detected in all four surveyed cities. In contrast, other genotypes demonstrated varying geographical patterns. Genotypes HLJ-CP1 and SHWR1 were found in three cities, except for Harbin. Genotype A was observed in the cities of Suihua and Qitaihe, while genotype EbpC was found in Shuangyashan and Harbin. Exclusive to Suihua were the genotypes WR8, SDD5, and SHWR2 to SHWR17. Similarly, genotypes CHPM1, Henan-II, HNH-22, I, JLD-XI, CS-4, and SYSWR1 to SYSWR36 were distinctively detected in Shuangyashan. Lastly, genotypes BEB6, HNH-25, and QTHWR1 to QTHWR8 were isolated in Qitaihe (Table 2). Meanwhile, 10 samples demonstrated infections with mixed genotypes, specifically, two samples from Qitaihe displayed the D + A genotype, one from Harbin exhibited the D + EbpC genotype, one from Shuangyashan presented with the CS-4 + SYSWR36 genotype, and six samples from Suihua were infected with the D + A, D + HLJ-CP1, D + SHWR1, D + SHWR16, D + SHWR17, and SDD5 + WR8 genotypes (Table 2).

### Similarity analysis of novel genotypes of *E. bieneusi*

The investigation of 61 novel *E. bieneusi* genotypes revealed that genotype SYSWR21 exhibited the highest significant genetic diversity. This genotype varied by 10 bases from its closest relative, genotype SDD2. Following this, genotype SYSWR13 exhibited a 9-base difference from its most similar genotype WR7. Genotype SYSWR7 and SYSWR4 had an 8-base difference from the genotypes WR7 and A, respectively. Genotypes SYSWR2 and SYSWR3 differed from genotype WR7 by 7 bases, while SYSWR5 differed from genotype SDD2 by the same margin. Genotypes SYSWR10, SYSWR14, and SYSWR15 each differed from WR7 by 5 bases. Genotypes SYSWR6, SYSWR8, SYSWR9, SYSWR11, and SYSWR12 all differed from genotype WR7 by 4 bases. Moreover, the genotypes SHWR1, SYSWR17, and SYSWR33 differed from genotypes WR7, HLJ-CP1 and D by 3 bases, respectively (Table 3). The genotypes that remained showed minor alterations, differing from the known genotypes by only 1 or 2 bases (Table 4). Notably, this study also identified length variations in the ITS region of *E. bieneusi*, with genotypes QTHWR7, SYSWR28, SYSWR29, and SYSWR15 exhibiting 242 bp, and genotype SYSWR21 exhibiting 244 bp.

**Table 3:** The results of similarity analysis comparing novel *E. bieneusi* genotypes which existence of three or more base differences in the ITS region of the rRNA gene.

Genotype (no.)	Genotype^a^/Base substitution (Position)
SHWR1 (PP719220)	WR7/ G to A (13), A to G (19), T to C (20)
SHWR17 (PP719236)	HLJ-CP1/ T to C (93), T to C (113), T to C (153)
SYSWR33 (PP719278)	D/ T to C (14), C to T (63), T to C (126)
SYSWR6 (PP719251)	WR7/ G to A (13), A to G (19), T to C (20), A to G (124)
SYSWR8 (PP719253)	WR7/ G to A (13), A to G (19), T to C (20), A to G (47)
SYSWR9 (PP719254)	WR7/ G to A (13), A to G (19), T to C (20), C to A (76)
SYSWR11 (PP719256)	WR7/ A to G (10), G to A (13), A to G (19), T to C (20)
SYSWR12 (PP719257)	WR7/ G to A (13), A to G (19), T to C (20), G to A (195)
SYSWR10 (PP719255)	WR7/ A to G (3), G to A (13), A to G (19), T to C (20), A to G (85)
SYSWR14 (PP719259)	WR7/ G to A (13), A to G (19), T to C (20), G to A (56), A to G (181)
SYSWR15 (PP719260)	WR7/ G to A (13), A to G (19), T to C (20), A to G (92), A to / (221)
SYSWR2 (PP719247)	WR7/ A to G (10), G to A (13), A to G (19), C to G (33), A to G (85), A to G (191), A to G (210)
SYSWR3 (PP719248)	WR7/ A to T (10), G to A (13), A to G (19), A to G (85), A to G (124), A to G (191), A to G (210)
SYSWR5 (PP719250)	SDD2/ G to A (13), A to G (31), G to A (46), G to A (48), G to A (89), G to A (93), G to A (213)
SYSWR4 (PP719249)	A/ G to A (13), C to T (81), G to A (82), G to A (88), G to A (92), G to T (136), G to A (147), A to G (164)
SYSWR7 (PP719252)	WR7/ A to G (10), G to A (13), A to G (19), T to C (20), T to C (28), T to C (111), A to G (191), A to G (210)
SYSWR13 (PP719258)	WR7/ A to G (10), G to A (13), A to G (19), A to G (31), C to G (33), A to G (85), T to C (93), A to G (124), A to G (191)
SYSWR21 (PP719266)	SDD2/ G to A (13), / to T (33), G to A (46), G to A (48), C to T (82), G to A (89), G to A (93), G to A (113), G to A (137), C to T (138)

a Representing known genotypes which have the largest similarity with novel genotypes obtained in our study.

**Table 4:** The results of similarity analysis comparing novel *E. bieneusi* genotypes which existence of one or two base differences in the ITS region of the rRNA gene.

Known Genotype ^a^ (*n*)	Novel genotype/Base substitution (Position)
D (*n* = 33)	SHWR2/T to C (39); SHWR4/T to A (32); SHWR5/C to T (113); SHWR9/A to G (101); SHWR11/T to C (132); SHWR12/T to C (107); SHWR14/A to G (215); QTHWR2/T to C (205); QTHWR3/G to A (174); QTHWR5/G to A (202); QTHWR7/G delete (131); SYSWR1/G to A (192); SYSWR16/A to G (162); SYSWR17/T to C (9); SYSWR18/A to G (121); SYSWR19/A to G (155); SYSWR20/G to A (85); SYSWR23/A to G (94); SYSWR26/T to A (9); SYSWR27/A to T (121); SYSWR28/T to delete (6); SYSWR30/G to A (77); SYSWR31/C to T (64); SYSWR32/T to C (6); SYSWR34/T to C (171); SHWR3/ A to G (53), T to C (91); SHWR6/A to G (114), T to A (163); SHRW8/A to G (135), T to A (163); SHWR13/T to C (44), G to A (242); SHWR15/A to G (60), G to A (69); QTHWR4/G to A (82), T to C (203); SYSWR25/A to T (215), G to T (225); SYSWR29/T delete (9), A to G (114)
A (*n* = 1)	QTHWR6/G to A (122)
gorilla1 (*n* = 2)	QTHWR8/C to A (79); SYSWR24/G to A (193)
Peru8 (*n* = 2)	SHWR16/G to A (83), C to T (153); SYSWR36/G to A (112), T to C (235)
CHLJ-C2 (*n* = 1)	QTHWR1/T to C (175)
HX1-13 (*n* = 1)	SHWR10/C to T (97)
BEB6 (*n* = 1)	SYSWR35/C to T (135)
NX3 (*n* = 1)	SYSWR22/T to C (14), G to T (70)
SDD5 (*n* = 1)	SHWR7/G to A (224)

a Representing known genotypes which have the largest similarity with novel genotypes obtained in our study.

### Phylogenetic relationship of *E. bieneusi* genotypes

**Figure 3:**
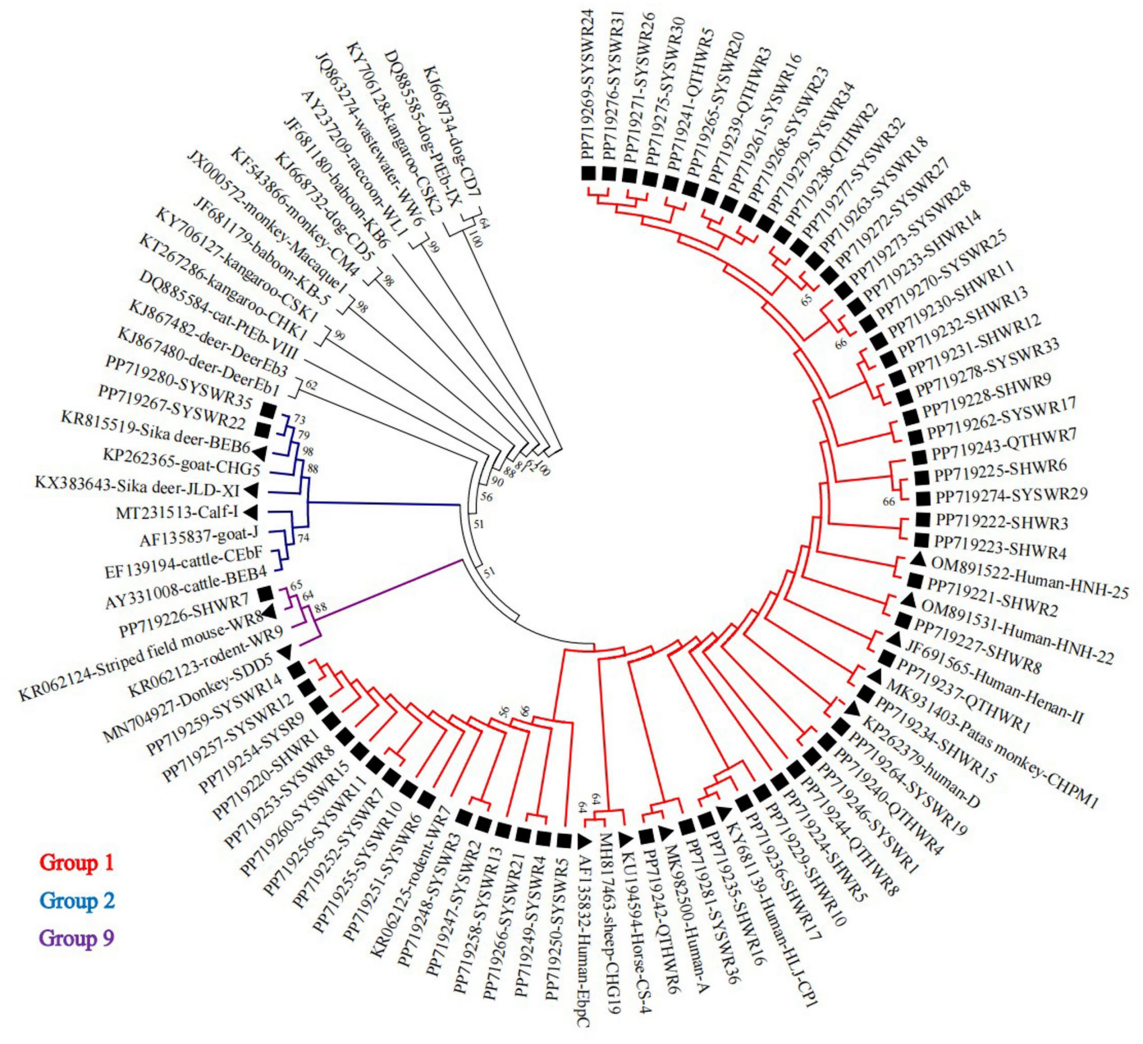
A phylogenetic tree was constructed, utilizing the Kimura-2-parameter model and the neighboring-joining method, to depict the genetic relationships among diverse *E. bieneusi* genotypes based on their ITS sequences. To ensure the reliability of the tree, bootstrap values were computed from 1,000 replicates. In this tree, genotypes are distinguished by triangle and squares filled in black, signifying known sequences and novel sequences identified in this study, respectively.

In a phylogenetic analysis conducted on a NJ tree of *E. bieneusi* genotypes, 61 novel genotypes were categorized into three distinct phylogenetic groups. Specifically, 58 genotypes were grouped into Group 1, alongside known genotypes A, CHPM1, CS-4, D, EbpC, Henan-II, HJL-CP1, HNH-22, and HNH-25. Two genotypes were allocated to Group 2, along with genotypes BEB6, JLD-XI, and I. Finally, genotype SHWR7 was assigned to group 9, alongside genotypes SDD5 and WR8. Figure 3 provides a visual representation of the specific results of this phylogenetic analysis, with an emphasis on the novel genotypes found in this investigation.

## Discussion

*Rattus norvegicus* is one of the most common species of rodents that have adapted well to anthropized ecosystems, present on all continents except Antarctica. These rats exploit various urban, rural, and sylvatic environments across diverse regions and climates. Their extensive distribution and the ease with which they adapt to various environments and resources might account for the abundance of zoonotic pathogens, including *E. bieneusi* (15). However, the occurrence of *E. bieneusi* in *R. norvegicus* has only been investigated in eight published investigations, all of which were conducted in China (11, 18–20, 22, 37). Except for Shanxi, which reported a slightly higher rate of 37.7% (18), all other studies reported infection rates that were lower than the 33.3% as observed in the present analysis. Even when compared to other rodent species, the present study’s infection rate remains high, given that the global average infection rate of *E. bieneusi* in rodents is 13.6% (15). Reported infection rates vary among rodent species, with higher rates in chipmunks (71.4%), prairie dogs (48.3%), striped field mice (42.9%), coypus (41.2%), and lesser rice-field rats (36.4%), while lower rates in bamboo rats (5.1%), pet chinchillas (3.6%), and muskrats (8.4%) [(15); (14)]. Poland has a greater prevalence of *E. bieneusi* infection in rodents compared to other countries (31). The endemicity levels of *E. bieneusi* infection in other countries are as follows: 8.8% in Iran (32), 21.1% in Spain (33), 18.0% in the United States (34), 10.7% in the border between the Czech Republic and Germany (35), 13.0% in Japan (38), and 1.1% in Slovakia (36). Overall, there is still limited knowledge about *E. bieneusi* infections in wild rodents worldwide. In general, the prevalence of *E. bieneusi* in different rodent species across various regions can be attributed to factors such as different rodent species, habitat environment, animal age, health status, population density of the hosts, and other unidentified variables of the animals tested. It is therefore recommended that further studies be conducted to elucidate the global prevalence of *E. bieneusi* in rodents, as this would aid in implementing effective public health interventions.

The present study has identified a remarkable degree of genetic diversity of *E. bieneusi* among *R. norvegicus*, revealing the presence of 75 genotypes. Of these, 14 have been previously characterized, while 61 are newly identified. It is worth mentioning that 10 of the identified genotypes, namely genotypes D, A, EbpC, HLJ-CP1, BEB6, Henan-II, HNH-22, HNH-25, CS-4 and I, have been previously documented in humans (3, 9). The 10 genotypes mentioned in this study make up 71.6% (202/282) of all the genotypes detected. Among these, genotype D is present in all four studied cities, suggesting its extensive dispersion and accounting for 59.2% (167/282) of the total genotypes. Furthermore, genotype D has garnered significant attention in numerous research, not only due to its presence among humans from 40 distinct nations but also because it has been detected in 63 animal hosts across 25 countries (3, 9). Additionally, its presence in certain water and vegetable samples indicates a broader ecological footprint (3). Considering the widespread occurrence of this genotype among rodents, it is not unexpected that it was found in all rodent species examined in this study. Overall, the present results provide further evidence that brown rats play a crucial role in the dissemination of *E. bieneusi*.

In the current study, genotype A emerged as the second most frequent genotype, trailing genotype D, with a frequency of 5.3% (15/282). This genotype has been identified to have a wide prevalence among individuals from several nations, including China, Cameroon, Gabon, Germany, Netherlands, Niger, Peru, Switzerland, and Thailand (12, 39). Genotype A has been sporadically found in animals such as baboons and dogs (40, 41). Importantly, this study presents the initial finding of this genotype in *R. norvegicus*, revealing its potentially vast host range. This discovery raises concerns about its potential transmission from *R. norvegicus* to humans, emphasizing the importance of conducting comprehensive surveys to gain a deeper understanding of its distribution and impact.

The current work found 12 *R. norvegicus* rats infected with the HLJ-CP1 genotype. This genotype was first found in a tumor patient and then detected in a wild rat from the same study region (13, 42). This observation strongly hints at the potential risk of interspecies and zoonotic transmission of this genotype within the same region. Consequently, it suggested that the genotype in tumor patients may have originated from rodents, and vice versa. Although EbpC was detected in only two *R. norvegicus*, this genotype was also identified to be widespread in native children, adult HIV patients, and multiple animal hosts, especially pigs (25, 26, 28, 43, 44). This further suggests the important role of rodents in the transmission of EbpC between humans and other animals.

Among the zoonotic genotypes identified, genotypes BEB6, Henan-II, HNH-22, HNH-25, CS-4 and I were only identified in a limited number of samples. These genotypes have been reported in only a few human cases from China. For instance, genotype BEB6 infected a child, Henan-II infected an HIV-positive patient, genotype I infected a child with diarrhea and an HIV-positive individual, while HNH-22 (OM891522) and HNH-25 (OM891531) were detected in humans residing in Hainan Province, China (39, 45–47). It is worth mentioning that, in addition to the genotype BEB6, additional genotypes were found in *R. norvegicus* and even rodents for the first time. This finding suggests that these genetic types may have a similar wide range of hosts, which requires more research to determine the exact source of human infection. As demonstrated in the present study, the existence of a novel host was verified, highlighting the need for continued research in this area.

No reported cases of human infections have been associated with the other genotypes (CHPM1, WR8, JLD-XI, and SDD5) identified in this study. The majority of these genotypes are restricted to certain animal hosts: for example, genotype JLD-XI exists in sika deer (48), genotype SDD5 (49) is identified in donkeys, and genotype CHPM1 is found in Patas monkeys (50). This study observed the presence of these genotypes in *R. norvegicus*, indicating a potentially significant role of these small rodents in the dissemination of *E. bieneusi* among wildlife, the environment, and domesticated animals. However, there remains uncertainty regarding the host range of these genotypes and the probability of zoonotic transmission. While this study offers novel insights, more thorough research is necessary to fully comprehend these problems.

A total of 61 previously novel genotypes were discovered in this investigation, which is quite high. Out of these genotypes, 54.1% (33/61) exhibited a difference of only one or two bases compared to genotype D (Table 4), suggesting that they may have evolved from genotype D. Therefore, they may possess a broad host range and a high potential for zoonotic transmission, similar to genotype D. Moreover, the evolutionary analysis revealed that nearly all of the novel genotypes, excluding SHWR7, belonged to either Group 1 or 2, with a significant preponderance of 95.1% (58/61) belonging to Group 1, indicating a potential for zoonotic transmission. However, it is becoming increasingly apparent that depending only on ITS sequence data is insufficient to provide a strong phylogenetic signal across the entire tree (9). Therefore, future studies necessitate the utilization of additional genetic markers to comprehensively understand the genetic affinities among these genotypes. Additionally, conclusive evidence of cross-species and zoonosis transmission requires further study.

## Conclusion

The current study has uncovered a high infection rate of *E. bieneusi* in *R. norvegicus* in Heilongjiang Province, China. Notably, genotyping analysis revealed an exceptionally diverse genotype composition, consisting of 14 known genotypes and 61 novel genotypes. Remarkably, zoonotic genotypes constituted a significant share of 71.6 and 98.4% novel genotypes were classified into Group 1 and Group 2. These findings underscore the crucial role of *R. norvegicus* in the transmission of *E. bieneusi* and highlight the potential public health risks in this region. Therefore, it is crucial to increase awareness regarding the hazards presented by these creatures and establish strategies to reduce their abundance, thereby minimizing environmental contamination. Additionally, the identification of 61 novel genotypes expands the understanding of the vast genetic diversity present in *E. bieneusi*. Further studies are necessary to better comprehend the genetic variety of the parasite and uncover its secrets.

## Data availability statement

The datasets presented in this study can be found in online repositories. The names of the repository/repositories and accession number(s) can be found in the article/supplementary material.

## Ethics statement

The animal study was approved by the National Institute of Parasitic Diseases, Chinese Center for Disease Control and Prevention, China (Reference no. IPD-2021-21). The study was conducted in accordance with the local legislation and institutional requirements.

## Author contributions

YJ: Data curation, Formal analysis, Funding acquisition, Investigation, Methodology, Project administration, Resources, Writing – original draft, Writing – review & editing. SZ: Formal analysis, Investigation, Methodology, Writing – original draft, Writing – review & editing. ZY: Formal analysis, Methodology, Visualization, Writing – original draft, Writing – review & editing. XH: Investigation, Methodology, Writing – original draft, Writing – review & editing. ZL: Formal analysis, Investigation, Methodology, Writing – original draft, Writing – review & editing. YW: Formal analysis, Methodology, Writing – original draft, Writing – review & editing. YS: Conceptualization, Resources, Supervision, Writing – original draft, Writing – review & editing. JC: Conceptualization, Resources, Supervision, Validation, Writing – original draft, Writing – review & editing.

## Footnotes

^1^http://www.megasoftware.net/

^2^http://www.megasoftware.net/
